# Support system networks: how support systems shape problematic social media use, mental health, and substance use in Czech adolescents

**DOI:** 10.1186/s13034-026-01081-w

**Published:** 2026-04-05

**Authors:** Jakub Helvich, Lukas Novak, Zdenek Meier, Martin Heveri, Peter Tavel

**Affiliations:** Olomouc University Social Health Institute, Palacký University Olomouc, Univerzitní 244/22, Olomouc, 771 11 Czech Republic

**Keywords:** Problematic social media use, Mental health, Substance use, Social support, Network analysis, HBSC

## Abstract

**Background:**

Problematic social media use (PSMU) has become a critical public health issue, with adolescents being one of the most engaged demographic. However, little is known about how distinct social support systems shape the associations between PSMU, mental health, and substance use.

**Objective:**

The first objective was to compare how support systems (peer, classmate, teacher, family, and self) shape the associations between PSMU, mental health, and substance use. The second objective was to explore which support systems are most prominent among boys and girls.

**Methodology:**

A representative sample of 5,487 Czech adolescents (50.88% boys, mean age = 15.48, SD = 0.53) was used for the analysis from the 2022 Health Behaviour in School-aged Children study. A network analysis based on undirected Mixed Graphical Models was used to examine the associations. Separate networks for each support system, stratified by gender, were estimated and compared.

**Results:**

Teacher and classmate support altered the associations in boys, while peer support was more prominent for girls. Family support altered both networks, though its function varied between genders. Self-support functioned as a bridge symptom in both genders, connecting PSMU with mental health and well-being, particularly in girls. In boys, classmate and family support made the association between PSMU and heated tobacco use as well as irritability absent, whereas in girls, peer and family support shaped the links between PSMU and well-being.

**Conclusion:**

The findings highlight the gender-specific nature of support systems and underscore the need for more nuanced approaches when facilitating external and internal support in adolescents.

**Supplementary Information:**

The online version contains supplementary material available at 10.1186/s13034-026-01081-w.

## Introduction

Problematic social media use (PSMU) is characterised by a maladaptive pattern of online behavior in which engagement with social media platforms interferes with everyday functioning ([95, 96]. While even frequent social media use may be normative [37, 72], PSMU is described by compulsive features resembling behavioral addictions. These include persistent thoughts about social media, diminished control over time spent online, and using platforms as a primary strategy for mood regulation or coping with negative emotions [77]. Over time, individuals may escalate their use to attain the same emotional relief and experience repeated unsuccessful efforts to cut back [77].

The prevalence of PSMU is especially pronounced among adolescents, a sociodemographic group undergoing critical developmental transitions while facing unique psychosocial pressures [57]; According to a recent WHO report, 11% of adolescents across 44 European and Central Asian countries now show signs of PSMU, up from 7% in 2018, with girls being more affected (about 13%) than boys (9%) [12, 109, 110]. These rising trends among adolescents are concerning, since PSMU also carries a range of significant mental health and other behavioural impairments.

PSMU has been consistently associated with higher levels of stress, anxiety, and depression [32, 95, 99, 114]. Previous studies also indicate that adolescents who show signs of PSMUoften experience poorer sleep quality [102], leading to chronic tiredness, low mood, and irritability [4, 80, 84], all of which can further exacerbate their mental health. Likewise, worse working memory capacity, attention control, and executive functioning also show a strong link to PSMU [48, 79, 88]. Additionally, recent evidence indicates positive links between PSMU and substance use, including alcohol, cannabis, and tobacco products [16, 36, 87], with younger individuals being predominantly at-risk [41]. Nevertheless, despite the growing body of research, there still remain several major research gaps on how these links vary when distinct support systems are considered.

Previous studies have repeatedly provided strong evidence of how social support might shape these associations. Strong family social support in particular has been associated with lower PSMU and fewer mental health symptoms [60, 95]. Additionally, better classmate support, teacher support, and overall school connectedness has been found to be associated with better mental health and reduced engagement in risk behaviours [11, 26, 70]. However, multivariate evidence indicates that supportive friendships can co-occur with higher risk behaviour once family and school supports are modelled, and research shows that parent support and peer support can operate in opposing directions within the same analysis [76, 112]. This shows that different support systems can co-occur or even compete statistically, obscuring unique associations.

By extension, resilience framework differentiates promotive resources (external supports) from promotive assets (internal competencies) and specifies compensatory versus modulating pathways by which these variables may alter risk relationships [19, 119]. Longitudinal evidence links internal supportive processes to later mental health, indicates that self-processes can mediate pathways from social contexts to mental health, and shows that self-supportive factors can directly increase risk of problematic digital behaviours even when broader external support systems show weaker prospective effect of PSMU [62, 103]. Collectively this means that internal assets are non-redundant with external resources.

Moreover, susceptibility to PSMU and its associated psychological harms differ by gender, with studies consistently showing higher prevalence and psychosocial vulnerability during this period among girls compared to boys [12, 105]. And although previous studies indicate that both external and internal support systems are beneficial for both girls and boys, gender can moderate how social support functions. Previous findings suggest boys benefit more from a better school climate, while girls can benefit more from strong family and peer relationships [3, 81, 108].

Although the present findings provide a strong foundation, there remain several critical gaps that previous studies have not addressed yet. First, previous studies have mostly explored support systems (e.g., family, teachers, peers, or classmates) in isolation rather than comparing their role in one statistical model [18, 63, 67, 73], leaving their potentially contrasting influence on PSMU, mental health, and substance use relations unexamined. Second, there remains a lack of fine-grained evidence on how distinct support systems shape item-level links between mental health symptoms, PSMU behaviours, and various substances. Third, comparisons between external and internal support systems, such as self-efficacy or self-competence, have largely remained overlooked. As a result, it remains unclear how adolescents’ own resilience stands in contrast to other external support domains. Finally, very few studies have explored gender-specific item-level patterns in how different support systems comparatively alter the associations between PSMU, mental health, and substance use.

Therefore, the objectives of the study are to compare how individual support systems (peer, classmate, teacher, family, and self) shape the associations between PSMU, mental health, and substance use in adolescents using item-level network analysis. Additionally, the study aims to explore which support system is most prominent in the networks of boys and girls.

## Methodology

### Participants and procedure

A nationally representative dataset of Czech adolescents was used as part of the 2022 Health Behaviour in School-aged Children (HBSC) study. Each study follows a standardised, internationally developed research protocol to ensure comparability across all participating countries [75]. Data collection took place between May and June 2022. Schools were randomly chosen, considering the principles of probability-proportional-to-size based region and school type. Of the 272 schools contacted, 246 agreed to participate in the study, resulting in a school response rate of 86.1%. A class was randomly selected from each of the 5th, 7th, and 9th grades (generally corresponding to ages 11, 13, and 15), making up a total sample of 14,879 pupils. Surveys were administered via Computer-Assisted Web Interviewing (CAWI) using the Unipark/TIVIAN platform and supervised by trained research assistants (*n* = 86) without the presence of teachers to minimise response bias. Students were allotted one standard school lesson (45 min) to complete the questionnaire. Participation was fully anonymous and entirely voluntary.

An additional 111 were removed due to unreliable responses, such as mutually exclusive or nonsensical answers to open-ended questions, and 12 respondents were discarded due to excessive missing data. Additionally, a total of 168 respondents were removed from the sample due to age outside the permitted range derived from the nationally validated birth date ranges for each grade collectively spanning from September 1, 2005 - August 31, 2012. Grades 5 and 7 were excluded from the final dataset, as these cohorts were not administered the extended questionnaire battery on substance use (such as cannabis, nicotine pouches or heated tobacco). The resulting final sample consisted of 5487 pupils (50.88% boys, mean age = 15.48, SD = 0.53).

### Measures

Internal consistency (McDonald’s omega) is reported exclusively for the support system measures, as these were aggregated into composite scores for the analysis. Reliability metrics are not reported for the PSMU, mental health, and well-being scales, as these were conceptualized and modeled as distinct item-level nodes within the network framework rather than as reflective latent constructs (Fried, 2020).

*The Social Media Disorder Scale (SMDS)* is a brief self-report measure designed to assess a range of symptoms associated with PSMU [107]. It includes nine yes/no items reflecting core addiction-related experiences, such as obsessive thinking, needing more time online, withdrawal symptoms, or interpersonal conflicts. The scale showed strong structural validity in adolescent populations across all 44 HSBC countries with good internal consistency (minimum alpha = 0.840) and supported cross-national measurement invariance [10]. In our study, we used the validated Czech version of the SMDS [93]. Participants respond based on their experiences over the past 12 months. The total score ranges from 0 to 9, with higher values indicating greater severity of PSMU.

In addition to the SMDS, a separate single-item question was included to assess whether participants prefer expressing their emotions via social media rather than through face-to-face interactions. This item was self-reported using a five-point Likert scale ranging from “Strongly Disagree” (1) to “Strongly Agree” (5).

*The Multiple Health Complaints Measure* is a self-report instrument composed of eight items that assess both physical and psychological symptoms [24]. It includes four physical symptoms (headaches, stomach pain, back pain, and dizziness) and four psychological ones (low mood, nervousness, irritability, and trouble falling asleep). The present study focused exclusively on the psychological symptom items. Participants were asked how often they had experienced each symptom over the past six months, with response options ranging from “Rarely or never” (5) to “About every day” (1). In this study, the item scoring was reversed, so that a higher score means a more frequent occurrence.

*The World Health Organisation-Five Well-Being Index (WHO-5)* is a short self-assessment tool commonly used to evaluate individuals’ psychological well-being over two weeks. It includes five affirmatively worded items reflecting positive emotional states, energy levels, and engagement in everyday life. Participants respond using a six-point scale, with responses ranging from “at no time” (0) to “all of the time” (5). Higher scores indicate better perceived well-being.

*Health risk behaviour* was measured using six items capturing substance-related risk-taking tendencies. Participants were asked: *“How frequently (if at all) do you use the following products or substances?”* The listed substances included: (1) heated tobacco, (2) nicotine pouches, (3) chewing tobacco, (4) kratom, and (5) snus. Responses were recorded on a 7-point scale ranging from *“never”* (1) to *“every or nearly every day”* (7). Moreover, participants answered a four-item question assessing recent substance use: *“On how many days (if at all) have you [item] in the past 30 days?”* The items referred to: (1) smoking cigarettes, (2) using electronic cigarettes (excluding heated tobacco), (3) drinking alcohol, and (4) smoking cannabis. Each of these items was rated on a 7-point scale from *“never”* (1) to *“30 days or more”* (7).

*The Family Affluence Scale (FAS)* estimates the socioeconomic status of respondents [23]. It is based on reported household possessions and living conditions, including ownership of a car, presence of a dishwasher, number of bathrooms and computers in a household, whether the child has their own bedroom, and how often the family travels abroad for holidays. The total score ranges from 0 to 13, with higher scores indicating higher socioeconomic status.

*Peer Support* was measured by four self-report items assessing perceived emotional and instrumental support from friends [118]. Each item reflects a distinct aspect of peer support, including the extent to which the respondent feels that their friends are willing to help them, are reliable in times of need, are available for sharing personal joys and concerns, and can be approached to talk about problems. Respondents indicated their agreement with each statement on a seven-point Likert scale, ranging from “Strongly disagree” (1) to “Strongly agree” (7). Higher scores indicate stronger perceived peer support. The internal consistency of the peer support measure was excellent: McDonald’s ω = 0.94.

*Family Support* was evaluated using four self-report items designed to assess respondents’ perceptions of emotional and instrumental support received from their family [118]. The items evaluate key aspects of familial support, including the extent to which they feel that their family is willing to help them, provides the emotional support they need, offers a safe space for discussing personal problems, and is willing to help them in making important decisions. Respondents indicated their agreement with each statement using a seven-point Likert scale, ranging from “Strongly disagree” (1) to “Strongly agree” (7). Higher scores reflect stronger perceived family support. The internal consistency of the family support measure was excellent: McDonald’s ω = 0.95.

*Classmate Support* was measured by three self-report items assessing respondents’ perceptions of the social climate and peer acceptance within their classroom, including the extent to which classmates enjoy spending time together, demonstrate kindness and helpfulness, and accept one another as they are. Participants rated their agreement with each statement on a five-point Likert scale ranging from “Strongly agree” (1) to “Strongly disagree” (5). The item scoring was reversed so that higher scores indicate higher perceived classroom peer support. The internal consistency of the classmate support measure was acceptable: McDonald’s ω = 0.79.

*Teacher Support* was assessed by three items designed to evaluate participants’ perceptions of emotional support provided by their teachers. The items capture respondents’ sense of being accepted by their teachers, the belief that their teachers genuinely care about them as individuals, and the degree of trust they place in their teachers. Respondents rated each statement on a five-point Likert scale ranging from “Strongly agree” (1) to “Strongly disagree” (5). The item scoring was reversed so that higher scores indicate higher perceived teacher support. The internal consistency of the teacher support measure was good: McDonald’s ω = 0.82.

*Self-Support* is a self-report composite encompassing six items designed to assess respondents’ perceived self-efficacy and self-control. Two items assess self-efficacy, including one’s perceived ability to solve problems when trying hard and to complete tasks once decided upon. Four additional items capture perceived control and coping, including the extent to which adolescents feel they can handle personal difficulties, that things are going as they wish, and whether they feel confident to influence important aspects of their lives through their effort. All items are rated on a five-point scale, with response options ranging from “Never” (1) to “Very often” (5). For analytic consistency, two negatively worded items were reverse-coded so that higher values uniformly reflect stronger self-support. The internal consistency of the self-support composite measure was acceptable: McDonald’s ω = 0.74.

### Data analysis

A network psychometrics approach was chosen over traditional latent variable modeling (e.g., SEM) because it theoretically conceptualizes PSMU, mental health, and substance use not as passive indicators of underlying disorders, but as a complex system of mutually interacting behaviors and symptoms [15]. This allows for an exploratory, item-level examination of how specific support systems bridge or alter the ecosystem of adolescent behaviors. Because cross-sectional data limits the ability to establish definitive causal directionality, especially among variables known to feature reciprocal feedback loops (e.g., low mood and social media escapism), we opted for an undirected network approach, which maps robust conditional dependencies without forcing unwarranted causal pathways.

Specifically, because the dataset comprised both categorical and continuous variables, network estimation was conducted using Mixed Graphical Models (MGM) grounded in the Markov Random Field (MRF) framework. In this analytical framework, each variable is depicted as a node, while edges represent conditional dependencies between variable pairs after controlling for all remaining nodes in the network [30]. The MRF approach was selected due to its ability to: (1) estimate all edges simultaneously while accounting for the rest of the network [28]; (2) capture bidirectional and reciprocal associations, including potential feedback mechanisms [22]; and (3) model intricate relationships at the level of single items, thereby allowing the identification of key nodes and the examination of network reconfiguration following the inclusion of new variables [30].

MGM estimation proceeds via generalised node-wise regression, where each variable is predicted by all others in turn [46]. A connection is considered present only if it is confirmed in both regressions involving the same pair of nodes, following the “AND” rule. To improve model parsimony and suppress spurious edges, Least Absolute Shrinkage and Selection Operator (LASSO) regularisation combined with the Extended Bayesian Information Criterion (EBIC) was used, with the parameter γ fixed at 0.5, yielding a conservative network structure that favours substantive over incidental links [29, 64]. Networks were visualised using the Fruchterman–Reingold spring layout, which optimises spatial arrangement by balancing node attraction and repulsion [34]. To ensure consistent interpretability, node positions were held constant across networks for direct visual comparison between groups.

Node predictability was quantified using *R²* for continuous nodes and classification accuracy for categorical ones, reflecting how much of each variable’s variance could be explained by its immediate neighbours [17]. Expected influence was computed to assess each node’s cumulative connectivity strength and overall contribution to the network’s structure. The robustness of edge weights and centrality indices was evaluated via the bootnet package [30], employing 2,500 non-parametric bootstrap iterations for edge-weight stability [13, 106] and 2500 case-dropping bootstrap samples for centrality stability assessment [30]. An assessment of missingness using Little’s MCAR procedure indicated that the data were missing at random. Consequently, observations containing incomplete information were removed prior to the analyses through listwise deletion.

Altogether, twelve networks were estimated: one for each support system, plus gender-specific networks. Network equivalence across estimated networks was assessed using the NetworkComparisonTest package [14]. Based on 1000 permutation resamples, two types of comparisons were performed: (1) network structure invariance, assessing whether the configuration of edge weights differed, and (2) global strength invariance, testing for differences in the overall magnitude of connectivity.

## Results

### Descriptive statistics

See Table 1 for descriptive statistics of problematic social media use and preference to discuss feelings online by gender, and Table 2for descriptive statistics of support systems and detected gender differences across different support systems.

**Table 1:** Descriptive characteristics of problematic social media use and preference to talk about feelings online in Czech adolescents

Social media disorder scale (SMDS) items	Boys (*N* = 2792)	Girls (*N* = 2695)
SM1		
No	2149 (77.0%)	1916 (71.1%)
Yes	465 (16.7%)	678 (25.2%)
Missing	178 (6.4%)	101 (3.7%)
SM2		
No	2230 (79.9%)	2115 (78.5%)
Yes	378 (13.5%)	477 (17.7%)
Missing	184 (6.6%)	103 (3.8%)
SM3		
No	2204 (78.9%)	2018 (74.9%)
Yes	401 (14.4%)	570 (21.2%)
Missing	187 (6.7%)	107 (4.0%)
SM4		
No	1983 (71.0%)	1536 (57.0%)
Yes	617 (22.1%)	1050 (39.0%)
Missing	192 (6.9%)	109 (4.0%)
SM5		
No	2200 (78.8%)	1991 (73.9%)
Yes	398 (14.3%)	595 (22.1%)
Missing	194 (6.9%)	109 (4.0%)
SM6		
No	2224 (79.7%)	2101 (78.0%)
Yes	369 (13.2%)	471 (17.5%)
Missing	199 (7.1%)	123 (4.6%)
SM7		
No	2302 (82.4%)	2165 (80.3%)
Yes	299 (10.7%)	418 (15.5%)
Missing	191 (6.8%)	112 (4.2%)
SM8		
No	1640 (58.7%)	947 (35.1%)
Yes	953 (34.1%)	1639 (60.8%)
Missing	199 (7.1%)	109 (4.0%)
SM9		
No	2297 (82.3%)	2168 (80.4%)
Yes	303 (10.9%)	414 (15.4%)
Missing	192 (6.9%)	113 (4.2%)
SMP		
Strongly disagree	920 (33.0%)	668 (24.8%)
Disagree	473 (16.9%)	441 (16.4%)
Neither agree nor disagree	603 (21.6%)	719 (26.7%)
Agree	384 (13.8%)	471 (17.5%)
Strongly agree	248 (8.9%)	313 (11.6%)
Missing	164 (5.9%)	83 (3.1%)

See Supplementary material 1 for an extended table of descriptive characteristics for other measures that were used in the study

SM1: can’t think of anything else but using social media; SM2: felt dissatisfied because wanted to use social media; SM3: felt bad when could not use social media; SM4: failed to spend less time on social media; SM5: lost interest in other activities because of social media use; SM6: had arguments with others because of social media use; SM7: lied to others about the amount of time spend on social media; SM8: used social media to escape negative feelings; SM9: had serious conflict with family because of social media use; SMP: prefers to talk about one’s own feelings online

**Table 2:** Descriptive statistics and socio-demographic comparison of support systems

Variable	*N* (%)	Peer support GD	Teacher support GD	Classmate support GD	Family support GD	Self-support GD	Peer support M(SD)	Teacher support M(SD)	Classmate support M(SD)	Family support M(SD)	Self-support M(SD)
Gender											
Boys	2792 (50.88)	*p* < 0.001	*p* < 0.001	*p* < 0.001	*p* < 0.001	*p* < 0.001	20.31 (6.69)	9.81 (2.87)	22.04 (6.66)	10.44 (2.8)	20.92 (3.64)
Girls	2695 (49.12)						21.09 (6.6)	9.39 (2.7)	20.04 (7.15)	9.86 (2.75)	18.85 (3.79)

For two-group comparisons the following tests were used via psychtoolbox [83] in R: Yuen’s test and Mann-Whitney U test

GD: group difference; M: mean; SD: standard deviation

### Network analysis

**Figs. 1 and 2:**
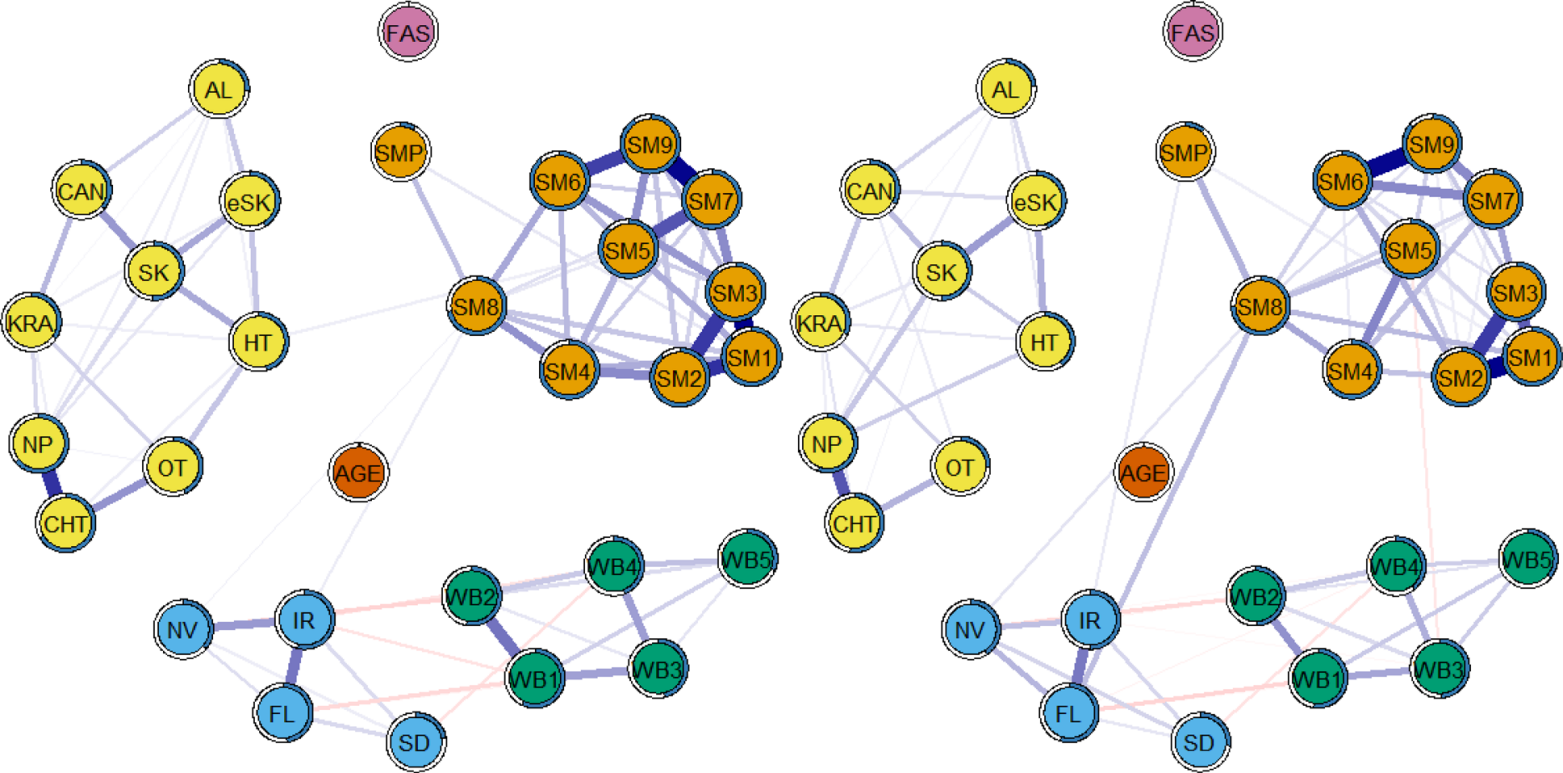
Boy (*n* = 2168) and girl (*n* = 2218) sample networks adjusted for age and FAS with expected influence centrality results. Thicker edges represent stronger associations. Blue edges reflect positive associations, while red edges represent negative associations. A higher degree of blue color inside the rings around nodes reflects higher node predictability. SM1 - SM9: SMDS items; SMP: Preference to talk about feelings online; WB1 - WB5: WHO-5 items; FAS: Family Affluence Scale; AGE: age; SD: sleeping difficulty; FL: feeling low; NV: nervousness; IR: irritability; AL: alcohol; SK: smoking tobacco; eSK: e-cigarette smoking; HT: heated tobacco; OT: oral tobacco; CHT: chewing tobacco; NP: nicotine pouches; KRA: kratom; CAN: cannabis

**Figs. 3 and 4:**
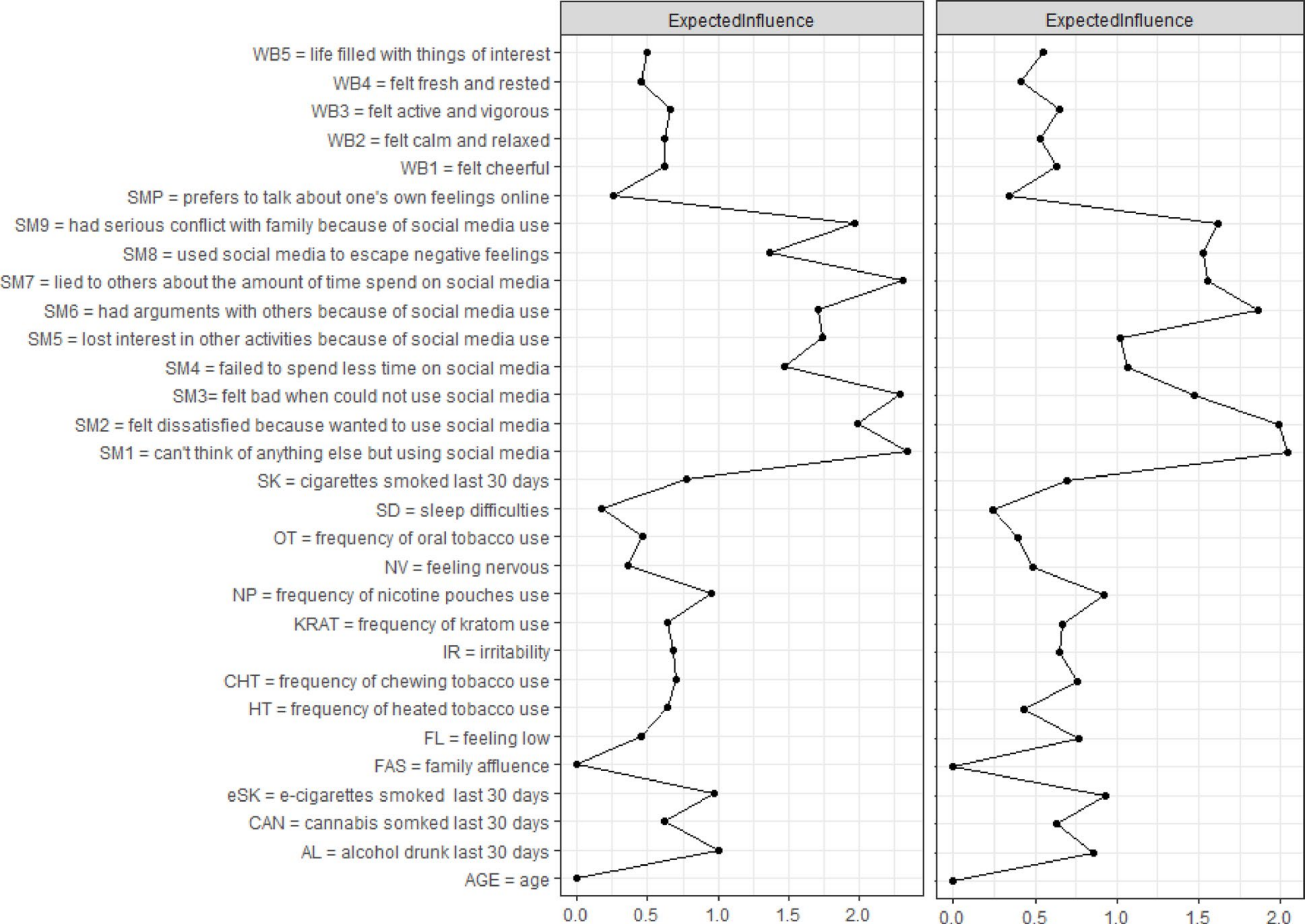
Expected influence centrality results for boys and girls. The X-axis in the expected influence graph shows how strongly a node is connected to others and how much impact it has on the network

In the first step, gender-stratified networks adjusted for age and FAS were estimated and subsequently compared (Figs. 1 and 2). Both networks revealed several positive associations (i.e., non-zero-weight edges) between PSMU and mental health complaints, with subtle gender-specific variations. Among boys, significant yet weak associations were observed between SM8 (“using social media to escape negative feelings”) and irritability (*w* = 0.04) as well as nervousness (*w* = 0.06). In girls, SM8 showed strong links with feeling low (*w* = 0.19) and nervousness (*w* = 0.08). Additionally, another significant connection was observed between SMP (“preference to talk about feeling online”) and feeling low (*w* = 0.06) in girls. In the boy network, PSMU, specifically SM5 (“losing interest in hobbies or other activities due to social media use”), was linked to heated tobacco use (*w* = 0.05). In contrast, no such associations between PSMU and substance use were found among girls. Instead, in the girl network, SM5 was negatively related to WB3 (“feeling active and vigorous”) (*w* = 0.07), whereas no such relationships between PSMU and well-being were detected in boys. Overall, most non-zero-weight edges connecting individual item clusters were relatively weak. The nodes with the highest expected influence centrality in boys were SM1, SM3, and SM7 and in girls were SM1, SM2, SM6, whereas the least central node was SD in both networks (Figs. 3 and 4). Detailed edge stability metrics for each gender-specific network are presented in the Supplementary materials 2 and 3.

### Differential role of peer support in the networks

**Figs. 5 and 6:**
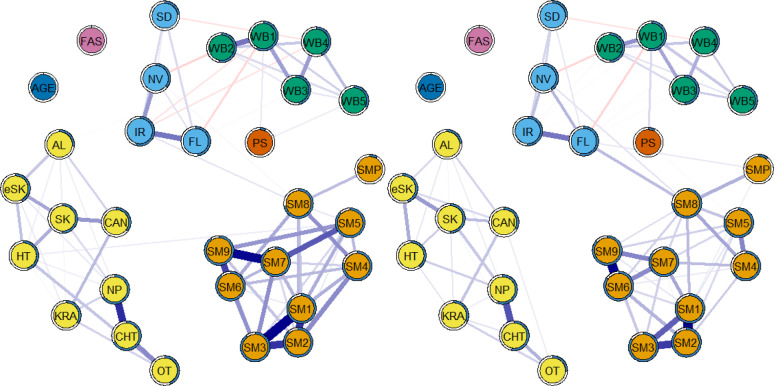
Boy (*n* = 2150) and girl (*n* = 2206) sample networks, controlling for peer support and adjusted for age and FAS. Thicker edges represent stronger associations. Blue edges reflect positive associations, while red edges represent negative associations. A higher degree of blue color inside the rings around nodes reflects higher node predictability. SM1 - SM9: SMDS items; SMP: Preference to talk about feelings online; WB1 - WB5: WHO-5 items; FAS: Family Affluence Scale; AGE: age; SD: sleeping difficulty; FL: feeling low; NV: nervousness; IR: irritability; AL: alcohol; SK: smoking tobacco; eSK: e-cigarette smoking; HT: heated tobacco; OT: oral tobacco; CHT: chewing tobacco; NP: nicotine pouches; KRA: kratom; CAN: cannabis; PS: Peer support

Subsequently, separate gender-stratified networks were estimated while controlling for each internal and external support system. When peer support was controlled for, no substantial changes in edge weights between PSMU, mental health complaints, and substance use were observed in the boy network (Fig. 5). In the girl network (Fig. 6), however, the negative association between WB3 (“feeling active and vigorous”) and SM5 (“losing interest in hobbies or other activities due to social media use”) became absent.

### Differential role of classmate support in the networks

**Figs. 7 and 8:**
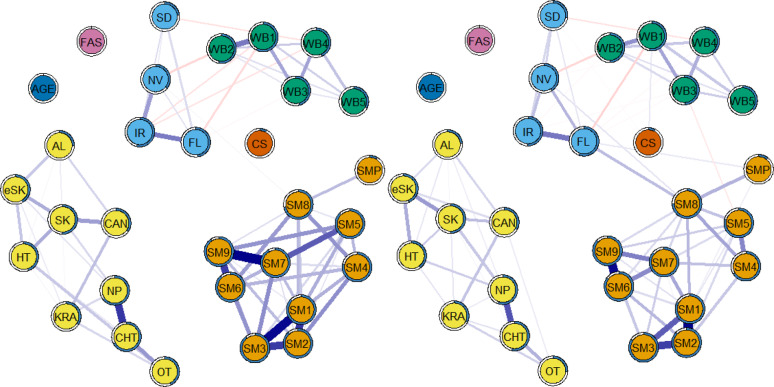
Boy (*n* = 2145) and girl (*n* = 2208) sample networks, controlling for classmate support and adjusted for age and FAS. Thicker edges represent stronger associations. Blue edges reflect positive associations, while red edges represent negative associations. A higher degree of blue color inside the rings around nodes reflects higher node predictability. SM1 - SM9: SMDS items: SMP: Preference to talk about feelings online: WB1 - WB5: WHO-5 items: FAS: Family Affluence Scale; AGE: age; SD: sleeping difficulty; FL: feeling low; NV: nervousness; IR: irritability; AL: alcohol; SK: smoking tobacco; eSK: e-cigarette smoking; HT: heated tobacco; OT: oral tobacco; CHT: chewing tobacco; NP: nicotine pouches; KRA: kratom; CAN: cannabis; CS: Classmate support

When classmate support was controlled for, more pronounced structural changes were observed in the boy network (Fig. 7). Specifically, the association between SM8 (“using social media to escape negative feelings”) and irritability was not observed once the classmate support node was included. Similarly, the previously observed link between SM5 and heated tobacco use also disappeared. In contrast, the inclusion of classmate support did not produce notable changes in the girl network (Fig. 8).

### Differential role of teacher support in the networks

**Figs. 9 and 10:**
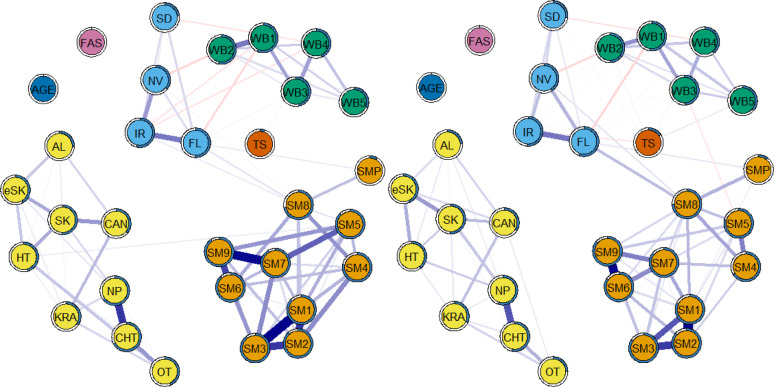
Boy (*n* = 2162) and girl (*n* = 2208) sample networks, controlling for teacher support and adjusted for age and FAS. Thicker edges represent stronger associations. Blue edges reflect positive associations, while red edges represent negative associations. A higher degree of blue color inside the rings around nodes reflects higher node predictability. SM1 - SM9: SMDS items; SMP: Preference to talk about feelings online; WB1 - WB5: WHO-5 items; FAS: Family Affluence Scale; AGE: age; SD: sleeping difficulty; FL: feeling low; NV: nervousness; IR: irritability; AL: alcohol; SK: smoking tobacco; eSK: e-cigarette smoking; HT: heated tobacco; OT: oral tobacco; CHT: chewing tobacco; NP: nicotine pouches; KRA: kratom; CAN: cannabis; TS: Teacher support

The inclusion of teacher support produced contrastive structural changes in the male network (Fig. 9). Specifically, new positive associations were detected between SM8 (“using social media to escape negative feelings”) and feeling low (*w* = 0.09), as well as between SMP (“preferring to talk about feelings online”) and feeling low (*w* = 0.06). In contrast, no notable structural changes were observed in the female network (Fig. 10) following the inclusion of teacher support.

### Differential role of family support in the networks

**Figs. 11 and 12:**
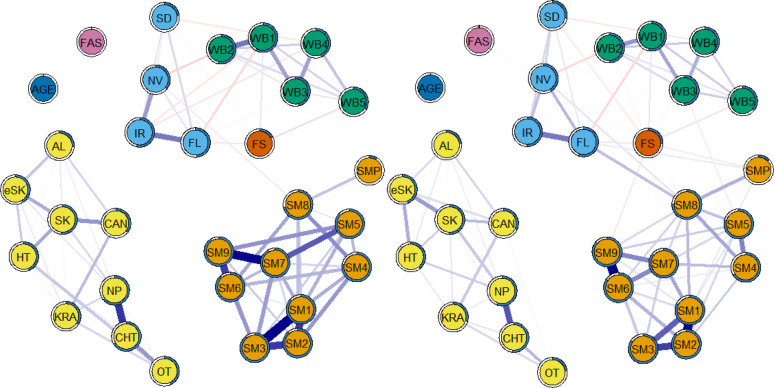
Boy (*n* = 2150) and girl (*n* = 2214) sample networks, controlling for family support and adjusted for age and FAS. Thicker edges represent stronger associations. Blue edges reflect positive associations, while red edges represent negative associations. A higher degree of blue color inside the rings around nodes reflects higher node predictability. SM1 - SM9: SMDS items; SMP: Preference to talk about feelings online; WB1 - WB5: WHO-5 items; FAS: Family Affluence Scale; AGE: age; SD: sleeping difficulty; FL: feeling low; NV: nervousness; IR: irritability; AL: alcohol; SK: smoking tobacco; eSK: e-cigarette smoking; HT: heated tobacco; OT: oral tobacco; CHT: chewing tobacco; NP: nicotine pouches; KRA: kratom; CAN: cannabis; FS: Family support

In comparison, family support had a more pronounced role in both networks. In boys (Fig. 11), the previously observed association between SM8 and irritability became absent, and the link between SM5 (“losing interest in hobbies or other activities due to social media use”) and heated tobacco use also disappeared once family support was added. Among girls (Fig. 12), family support showed a more central role, functioning as a bridge symptom (a node connecting previously distinct variable clusters). It interconnected the PSMU cluster (“family conflicts due to social media use” and “preference to talk about feelings online”) with both the mental well-being cluster (“cheerfulness”, “calmness”, “freshness”, and “fulfilment”) and the mental health complaints cluster (“feeling low” and “difficulty sleeping”).

### Differential role of self-support in the networks

**Figs. 13 and 14:**
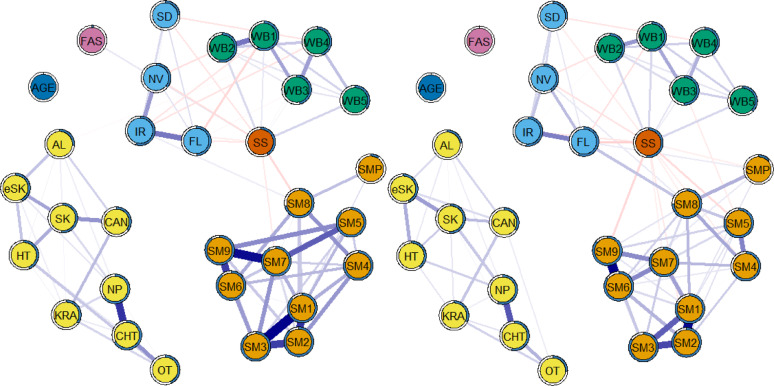
Boy (*n* = 2141) and girl (*n* = 2207) sample networks, controlling for self-support and adjusted for age and FAS. Thicker edges represent stronger associations. Blue edges reflect positive associations, while red edges represent negative associations. A higher degree of blue color inside the rings around nodes reflects higher node predictability. SM1 - SM9: SMDS items; SMP: Preference to talk about feelings online; WB1 - WB5: WHO-5 items; FAS: Family Affluence Scale; AGE: age; SD: sleeping difficulty; FL: feeling low; NV: nervousness; IR: irritability; AL: alcohol; SK: smoking tobacco; eSK: e-cigarette smoking; HT: heated tobacco; OT: oral tobacco; CHT: chewing tobacco; NP: nicotine pouches; KRA: kratom; CAN: cannabis; SS: Self-support

When self-support was introduced into both the boy (Fig. 13) and girl networks (Fig. 14), it exerted a dual alteration of their structures. First, the previously observed association between SM8 (“using social media to escape negative feelings”) and nervousness (in contrast to irritability in earlier models) became absent in both networks. Additionally, in boys, the link between SM5 (“losing interest in hobbies or other activities due to social media use”) and heated tobacco use also disappeared once self-support was controlled for.

Second, self-support emerged as a key bridge symptom in both networks. Among boys, it interconnected two PSMU items (“using social media to escape negative feelings” and “lying about the amount of time spent on social media”), with all mental health complaints and mental well-being items. In girls, the bridging effect was considerably stronger, linking a broader range of PSMU items (SM9, SM5, SM2, SM7, SM8, SM4, and SMP) with all mental health complaints and mental well-being items.

### Network comparison testing

The network structure invariance test revealed significant gender differences in network configuration for all support systems: (boy peer support x girl peer support: M = 0.42, *p* = 0.001; boy classmate support x girl classmate support: M = 0.43, *p* = 0.006; boy teacher support x girl teacher support: M = 0.41, *p* = 0.01; boy family support x girl family support: M = 0.42, *p* = 0.006; boy self-support x girl self-support: M = 0.41, *p* = 0.015) These results indicate that the pattern and strength of specific edges differ between boys and girls, suggesting that the relational structure among PSMU, mental health, and substance use is not comparable across gender within any support-system network. In contrast, the global strength invariance test showed mixed evidence regarding overall connectivity. For peer support, classmate support, and teacher support, boys and girls differed significantly in total network strength (S = 1.52, *p* = 0.014; S = 1.49, *p* = 0.018; S = 1.17, *p* = 0.05, respectively), indicating that one gender’s network is more densely connected in these domains. However, no significant differences were found for family support (S = 0.83, *p* = 0.18) or self-support (S = 0.75, *p* = 0.26), suggesting similar overall levels of connectivity for these two networks. For complete results, see Supplementary materials 4–8.

## Discussion

The objectives of this study were to examine how distinct support systems (peer, classmate, teacher, family, and self) shape the associations between PSMU, mental health, and substance use in adolescents. Furthermore, the study aimed to identify which support systems are most prominent among boys and girls. Teacher support and classmate support altered the relationships in the network of boys but not girls, while peer support showed a more pronounced role in the girl network. Family support altered the relationships in both girls and boys, but a gender-specific pattern was found. Self-support emerged as the most integrative variable for both girls and boys, functioning as a bridge symptom, linking the PSMU cluster with mental health and well-being item clusters, with a substantially stronger bridging effect observed in girls. In boys, classmate and family support produced notable structural changes in the networks, making several previously observed associations absent, specifically between social media escapism and irritability, and between social media–related disinterest in hobbies and heated tobacco use. In contrast, in girls, family support acted as a bridge symptom interconnecting a small subset of items across PSMU, mental health complaints and mental well-being clusters. When teacher support was controlled for in boys, new positive associations were detected between social media escapism and feeling low, as well as between preferring to talk about feelings online and feeling low. Among girls, controlling for peer support made the negative relationship between feelings of vigour and social media–related disinterest in hobbies absent.

Teacher support emerged as a critical variable for boys but not for girls. When teacher support was controlled for in the boys’ network, previously latent links became evident: social media escapism and preferring to talk about feelings online were positively associated with feeling low. Longstanding evidence suggests that strong teacher support is broadly linked to better adolescent well-being [43, 54, 101]. Previous research revealed that perceived teacher support accounted for a significant portion of variance in middle-school students’ life satisfaction [101]. However, girls often report lower perceived teacher care than boys, but both generally benefit from supportive teacher relationships [40]. This discrepancy underscores that different support systems may operate in distinct ways across genders. Prior studies focusing on multiple support sources suggest that adolescent girls often draw more emotional support from peers and family, whereas boys may have fewer outlets and thus stand to gain relatively more from regular positive teacher engagement at school [5, 21, 42].

One plausible explanation is rooted in gender differences in socialization and help-seeking. Research has consistently found that adolescent boys are less likely to actively seek emotional support or disclose problems than girls who also often report more dense social support systems [42, 98]. In this sense, teachers for boys might represent a uniquely trusted adult or mentor figure with whom they feel safe to share their mental struggles, especially if they are male [53, 89]. However, when teacher support was controlled for in the network, several previously hidden connections between PSMU and psychological distress emerged. This indicates that teacher support can compensate for certain vulnerabilities in boys. Therefore, even if a boy engages in PSMU, a supportive teacher-student relationship might prevent those behaviors from translating into severe mental health complaints. One reason might be that a supportive teacher might encourage or model healthier behaviors, self-control, bolster reliance, and promote face-to-face dialogue in a more controlled environment which might be more suited for boys [1, 9, 89]. This modulating change might also be attributed to the systematic nature of the school environment and the way boys socialize [91], which might also translate to their classmates.

Classmate support emerged as another key variable in adolescent boys’ networks, whereas its prominence was not observed in girls. Specifically, the association between social media escapism and irritability, as well as between social media-related disinterest in hobbies and heated tobacco use was no longer found in the network. Studies have long suggested that the impact of peer support may differ by gender [85, 92]. A longitudinal study by Rueger et al., [92] revealed that classmate support uniquely predicted better mental health and better adjustment in adolescent boys, but not in girls. At the same time, other support sources appear especially salient for girls, which may explain why broad classmate support did not alter girls’ network [52, 85]. Adolescent girls tend to gain protective benefits primarily from close intimacy and support in friendships, whereas boys often benefit from group belonging and acceptance [39, 82, 91].

Moreover, boys typically derive self-worth and stress relief from group camaraderie and shared activities [51, 91]. Therefore, in a supportive class environment, boys are likely to receive exactly what they most value, which might provide a substantial coping outlet for distress leaving them less prone to seek escape via digital means or substance use. By contrast, the same classmate support may feel more peripheral to girls’ core support needs. Another consideration is that boys, on average, report slightly lower willingness to seek emotional support and are socialized to appear self-reliant [31, 42, 98]. When support does come in a form that is congruent with male socialization it can indirectly improve emotional well-being without requiring boys to explicitly seek help [59, 98]. Altogether, this suggests that a supportive classmate environment is particularly critical when promoting healthier habits for boys.

On the contrary, peer support was a critical variable for girls. When peer support was accounted for, the previously observed negative association between girls’ feelings of vigor and energy and their social media–related disinterest in hobbies and leisure became absent. Research consistently shows that adolescent girls place greater emphasis on and derive more emotional input from their friendships, which can shape how they respond to challenges like PSMU [25, 47, 113]. Previous studies demonstrated that adolescent girls are more likely than boys to turn to friends for emotional support when distressed [100, 113], and not only do they seek friend support more, but also the emotional support from friends is more strongly linked to psychological well-being in girls than in boys [2]. Evidence likewise points to gender-specific pathways in how social support may shape online behavior. For instance, a study revealed that for girls, higher real-life social support was directly associated with lower risk of PSMU, whereas in boys, offline support related to PSMU only indirectly [2, 7]. This suggests that real life support has a more immediate and tangible role inshaping girls’ online habits.

One explanation for this finding might be that girls with strong peer support are more likely to feel more energetic, and those same supportive friends likely encourage or participate in offline hobbies, thereby keeping the girl interested in those activities. Indeed, studies show that supportive peers can provide motivation, co-engagement, and accountability for hobbies, thereby counteracting the tendency of PSMU to displace real-world interests [7, 78]. An alternative but complementary interpretation may be that girls who are naturally more vigorous and positive tend to form and maintain stronger friendships, and those friendships then help sustain their interest in diverse activities [6, 27]. Meanwhile, girls low in vigor, experiencing frequent tiredness or fatigue, might struggle to connect with peers or have a limited support network as they instead turn to solitary social media use to inactively supplement social bonds [58, 104, 115]. Such notions suggest that adolescent boys may derive resilience from a general sense of belonging or acceptance in school, whereas girls rely relatively more on close peer support.

The present study found that strong family support altered the network of PSMU and associated health outcomes in both boys and girls, albeit through distinct mechanisms. Numerous studies have shown that adolescents who feel supported by their families report fewer mental health problems, lower substance use, and even reduced risky behaviors across the board [33, 74, 97]. Supportive family environments have also been linked to lower risk of PSMU. For instance, in a multinational study, Lahti et al., [60] found that higher family support was associated with a lower likelihood of PSMU and better health outcomes. Although the protective value of social support is well established, previous studies have reported nuanced gender differences in how adolescents benefit from family support [49, 81]. Some research suggests that girls may rely on and benefit from family support to a greater extent than boys [3, 81]. This indicates that girls often benefit more for emotional outcomes, whereas boys may benefit on behavioral outcomes, though many effects are shared by both [74]. In adolescent boys, PSMU has been frequently tied to externalizing tendencies [65].

One disappearing connection in the present study was between PSMU and irritability. A supportive family with open family communication and shared family activities, provides healthier outlets for stress and emotional expression and may provide more opportunities for parents to detect problematic behaviours [8, 66]. This may similarly also explain the nullified link between social-media–related disinterest toward other hobbies and substance use. Supportive families tend to encourage involvement in alternative activities even if the children are disinterested at first [94]. This reduces the idle time and social isolation that often accompany both high screen time and substance use. Second, high family support often coexists with greater parental oversight and communication about risky behaviors. Parents who are emotionally supportive are also more likely to notice a child’s disengagement from hobbies or signs of substance use and intervene constructively [74, 90].

In girls, family support functioned rather as a bridge symptom connecting PSMU, mental health complaints, and well-being. Previous studies revealed that girls especially rely on family when experiencing distress and integrate their social support more deeply into their psychosocial functioning [52, 116]. This suggests that girls may be especially sensitive to the presence or absence of family emotional support, which in turn affects both their well-being, their online behaviour and how they cope.

Self-support emerged as a key bridge symptom linking PSMU to adolescent mental health and well-being outcomes. Additionally, this bridging role of self-support was substantially stronger in girls than in boys. Studies found internal assets to be more influential than external assets for well-being and youth development [38, 71, 111]. Specific to digital context, internal factors such as high self-control, self-esteem, or self-efficacy have consistently been linked to lower risk of PSMU [20, 55, 117]. This indicates that adolescents’ personal sense of agency may be the conduit through which PSMU correlates with their mental health.

The contrastive role of internal and external support likely lies in how these systems operate in adolescent development. Self-support is a proximal determinant of behavior and emotional health [44, 45]. An adolescent’s belief in their ability to handle challenges influences their coping choices and stress levels immediately and personally [35]. In contrast, external support systems exert their influence more distally, often by shaping that very self-belief or by providing help that youths must then internally utilize [50, 69].

However, the same role of internal support appears to be more integral for girls rather than boys. First, it is well-documented that adolescent girls report higher rates of PSMU and related mental health issues than boys [12, 61]. Researchers have attributed this gender gap to factors like cyberbullying victimization, social comparison, and body image concerns, which tend to affect girls more severely in online environments [58, 68]. All these factors that disproportionately affect girls often interplay with their internal systems, which may determine whether they cope adaptively or turn to problematic escapes like social media [20, 56, 86]. Therefore, self-support for girls is more integral for navigating the gender-specific challenges tied to PSMU.

### Strengths and limitations

 However, several limitations should be acknowledged. First, the cross-sectional research design restricts the interpretation of causality between examined variables. Using longitudinal or experimental methodologies in future studies could help reveal whether observed associations reflect directional influences, reciprocal relationships, or co-occurrence. Second, the use of self-reported measures introduces possible distortions, as respondents might misrepresent or inaccurately recall their social media habits or psychological well-being due to social desirability effects or recall biases. Third, the research sample was made up exclusively of the Czech adolescent population, which may reduce the extent to which findings apply to broader international settings. Cultural variation, differences in social expectations, and national policy contexts could shape patterns of mental health and social media usage differently elsewhere. Fourth, although network analysis is powerful for illustrating complex interrelations, it is limited in detecting non-linear dynamics that may exist within the data. Fifth, although the study focuses on problematic social media use, which captures qualitative aspects of engagement, time spent on social media may represent an important contextual factor influencing network relations and gender differences. Future research should examine how time spent and problematic use jointly operate within a network framework. Lastly, the study did not focus on specific subpopulations, such as youth from immigrant backgrounds or those identifying as LGBTQ+, who may be at greater risk for mental health concerns. Directly focusing on such groups in future work would improve the relevance and inclusivity of our findings.

### Implications

#### Implications for research

Given the cross-sectional design, our findings should motivate prospective research that explores temporal relationships. Future work could use multi-wave cohorts, ecological momentary assessment, and multilevel or time-varying network models to examine whether changes in peer, classmate, teacher, family, and self-support co-occur with reconfigurations in links between PSMU, mental health, and substance use. Because self-support consistently bridged clusters, trials, and longitudinal studies might test whether changes in intrapersonal resources coincide with modulations of high-risk connections, especially among girls. Studies should also model classroom and school climate using multilevel networks, evaluate support quality and offline versus online support, expand substance-use indicators, and test robustness via measurement invariance, DIF, and alternative regularisation choices. Additionally, it is essential to replicate our findings across diverse cultural and societal contexts to capture potential cross-national differences and ensure broader generalizability.

### Implications for practice

For practice, these patterns point to tailoring specific supports by gender and delivery setting. In boys, classroom climate initiatives and teacher-focused training may be especially useful, since classmate and teacher support were linked with changes in network relations. These findings suggest that teacher and classroom-based interventions may influence not only mental health directly but also the broader structure of links between PSMU, mental health, and substance use conjointly. Teaching staff should watch for co-occurring escapist social media use, irritability, low mood, and loss of interest in hobbies, and align digital well-being efforts with tobacco-prevention counselling. In girls, peer-led mentoring and group-based socio-emotional learning may be prioritised, as peer support showed influence on well-being. This indicates that peer-oriented approaches may be particularly relevant for modifying pathways through which PSMU relates to emotional functioning. Combining this with brief skills that improve self-support (for example, self-compassion, emotion regulation, and self-efficacy) may coincide with broader benefits, given the bridging role of self-support across clusters.

Because family support shaped relationships in both networks to varying degrees, schools and clinicians can offer parent guidance on constructive communication about online activity, managing conflict, and consistent but non-punitive rules. The observation that support variables alter network structure encourage multi-tiered approaches that combine universal support with targeted interventions. Implementing routine screening for escapist use and mood complaints, using a multi-tiered support system (universal education plus targeted follow-up), and ensuring interventions respect student autonomy, avoid pathologising normative use, and protect privacy could together provide far-reaching benefits for both boys and girls. This highlights the importance of supportive rather than overly surveillance-oriented approaches.

From a theoretical perspective, rather than acting solely as distal protective factors, peer, teacher, family, and self-support appear to shape how these constructs are interconnected. The prominent bridging role of self-support extends social support theory by highlighting the importance of intrapersonal regulatory processes in a media-saturated developmental context. Moreover, the observed gender-differentiated patterns suggest that contemporary adolescent development is characterised by multiple context-specific support configurations, refining traditional hierarchical models of support.

## Conclusions

This study aimed to explore how various forms of support, including peer, classmate, teacher, family, and self-support, shape the relationships between PSMU, mental health, and substance use among adolescents. It also aimed to determine which support systems are most prominent among boys and girls. In boys, both teacher and classmate support altered network relationships, while peer support had a stronger role in girls. Family support reshaped the structure of both networks, but gender-specific patterns emerged. Self-support proved to be the most interconnected support system overall, functioning as a key bridge that linked the PSMU item cluster with mental health and well-being clusters, with this role being much more prominent among girls. In boys, classmate and family support produced considerable structural changes, making several associations no longer detectable, specifically between social media escapism and irritability, and between social media–related disinterest in hobbies and heated tobacco use. In contrast, in girls, family support acted as a bridge symptom interconnecting a small subset of items across PSMU, mental health complaints and mental well-being clusters. When teacher support was controlled for in boys, new positive associations appeared between social media escapism and low mood, as well as between preferring to talk about emotions online and low mood. In girls, controlling for peer support eliminated the negative association between mental well-being and PSMU.

## Electronic Supplementary Material

Below is the link to the electronic supplementary material.

Supplementary Material 1.

Supplementary Material 2.

Supplementary Material 3.

Supplementary Material 4.

Supplementary Material 5.

Supplementary Material 6.

Supplementary Material 7.

Supplementary Material 8.

## Data Availability

Data, programming scripts, and additional resources linked to this research can be accessed in the Open Science Framework (OSF) repository via the following link: https://osf.io/m4k6d/overview.
