# Supplementary material for: Support system networks: how support systems shape problematic social media use, mental health, and substance use in Czech adolescents
Source: Child Adolesc Psychiatry Ment Health. 2026 Apr 5;20:78. doi: 10.1186/s13034-026-01081-w (PMC13235073; doi:10.1186/s13034-026-01081-w)
Supplement: Supplementary file 1 — Supplementary Material 1. [file 13034_2026_1081_MOESM1_ESM.docx]

Descriptive statistic of the study sample

| Variable | Boys  (N = 2792)  n(%) | Girls  (N = 2695)  n(%) | |
| --- | --- | --- | --- |
| IQOS |  |  | |
| 1. Never | 2058 (87.72) | 1900 (81.41) | |
| 2. Not in the last year | 65 (2.77) | 79 (3.38) | |
| 3. Once a year | 74 (3.15) | 108 (4.63) | |
| 4. Few times a year | 62 (2.64) | 93 (3.98) | |
| 5. 1–3 times a month | 38 (1.62) | 64 (2.74) | |
| 6. 1–4 times a week | 21 (0.9) | 41 (1.76) | |
| 7. Everyday or almost everyday | 28 (1.19) | 49 (2.1) | |
| chewtob |  |  | |
| 1. Never | 2049 (87.34) | 2137 (91.56) | |
| 2. Not in the last year | 71 (3.03) | 60 (2.57) | |
| 3. Once a year | 59 (2.51) | 49 (2.1) | |
| 4. Few times a year | 56 (2.39) | 30 (1.29) | |
| 5. 1–3 times a month | 26 (1.11) | 31 (1.33) | |
| 6. 1–4 times a week | 31 (1.32) | 7 (0.3) | |
| 7. Everyday or almost everyday | 54 (2.3) | 20 (0.86) | |
| kratom |  |  | |
| 1. Never | 2086 (88.92) | 2183 (93.53) | |
| 2. Not in the last year | 35 (1.49) | 19 (0.81) | |
| 3. Once a year | 81 (3.45) | 55 (2.36) | |
| 4. Few times a year | 65 (2.77) | 40 (1.71) | |
| 5. 1–3 times a month | 45 (1.92) | 17 (0.73) | |
| 6. 1–4 times a week | 9 (0.38) | 10 (0.43) | |
| 7. Everyday or almost everyday | 25 (1.07) | 10 (0.43) | |
| nicpouch |  |  | |
| 1. Never | 1915 (81.63) | 2034 (87.15) | |
| 2. Not in the last year | 80 (3.41) | 65 (2.78) | |
| 3. Once a year | 104 (4.43) | 70 (3) | |
| 4. Few times a year | 84 (3.58) | 64 (2.74) | |
| 5. 1–3 times a month | 51 (2.17) | 36 (1.54) | |
| 6. 1–4 times a week | 36 (1.53) | 25 (1.07) | |
| 7. Everyday or almost everyday | 76 (3.24) | 40 (1.71) | |
| snus |  |  | |
| 1. Never | 2219 (94.59) | 2279 (97.64) | |
| 2. Not in the last year | 23 (0.98) | 19 (0.81) | |
| 3. Once a year | 29 (1.24) | 11 (0.47) | |
| 4. Few times a year | 20 (0.85) | 8 (0.34) | |
| 5. 1–3 times a month | 16 (0.68) | 6 (0.26) | |
| 6. 1–4 times a week | 12 (0.51) | 3 (0.13) | |
| 7. Everyday or almost everyday | 27 (1.15) | 8 (0.34) | |
| alcohol |  |  | |
| 1. Never | 1272 (54.22) | 1213 (51.97) | |
| 2. 1-2 days | 637 (27.15) | 676 (28.96) | |
| 3. 3-5 days | 206 (8.78) | 267 (11.44) | |
| 4. 6-9 days | 106 (4.52) | 103 (4.41) | |
| 5. 10-19 days | 57 (2.43) | 38 (1.63) | |
| 6. 20-29 days | 17 (0.72) | 18 (0.77) | |
| 7. 30 days (or more) | 51 (2.17) | 19 (0.81) | |
| cannabis |  |  | |
| 1. Never | 2167 (92.37) | 2190 (93.83) | |
| 2. 1-2 days | 96 (4.09) | 76 (3.26) | |
| 3. 3-5 days | 24 (1.02) | 24 (1.03) | |
| 4. 6-9 days | 9 (0.38) | 10 (0.43) | |
| 5. 10-19 days | 12 (0.51) | 11 (0.47) | |
| 6. 20-29 days | 6 (0.26) | 3 (0.13) | |
| 7. 30 days or more | 32 (1.36) | 20 (0.86) | |
| esmok |  |  | |
| 1. Never | 1930 (82.27) | 1798 (77.04) | |
| 2. 1-2 days | 157 (6.69) | 193 (8.27) | |
| 3. 3-5 days | 77 (3.28) | 91 (3.9) | |
| 4. 6-9 days | 58 (2.47) | 47 (2.01) | |
| 5. 10-19 days | 44 (1.88) | 84 (3.6) | |
| 6. 20-29 days | 19 (0.81) | 31 (1.33) | |
| 7. 30 days or more | 61 (2.6) | 90 (3.86) | |
| smok |  |  | |
| 1. Never | 2064 (87.98) | 2003 (85.82) | |
| 2. 1-2 days | 100 (4.26) | 105 (4.5) | |
| 3. 3-5 days | 31 (1.32) | 48 (2.06) | |
| 4. 6-9 days | 27 (1.15) | 31 (1.33) | |
| 5. 10-19 days | 25 (1.07) | 39 (1.67) | |
| 6. 20-29 days | 22 (0.94) | 28 (1.2) | |
| 7. 30 days (or more) | 77 (3.28) | 80 (3.43) | |
| feellow |  |  | |
| 1. About every day | 202 (8.61) | 513 (21.98) | |
| 2. More than once a week | 261 (11.13) | 539 (23.09) | |
| 3. About every week | 348 (14.83) | 455 (19.49) | |
| 4. About every month | 526 (22.42) | 451 (19.32) | |
| 5. Rarely or never | 1009 (43.01) | 376 (16.11) | |
| irritable |  |  | |
| 1. About every day | 302 (12.87) | 567 (24.29) | |
| 2. More than once a week | 420 (17.9) | 707 (30.29) | |
| 3. About every week | 571 (24.34) | 512 (21.94) | |
| 4. About every month | 656 (27.96) | 420 (17.99) | |
| 5. Rarely or never | 397 (16.92) | 128 (5.48) | |
| nervous |  |  | |
| 1. About every day | 285 (12.15) | 726 (31.11) | |
| 2. More than once a week | 391 (16.67) | 613 (26.26) | |
| 3. About every week | 518 (22.08) | 426 (18.25) | |
| 4. About every month | 618 (26.34) | 380 (16.28) | |
| 5. Rarely or never | 534 (22.76) | 189 (8.1) | |
| sleepdificulty |  |  | |
| 1. About every day | 242 (10.32) | 391 (16.75) | |
| 2. More than once a week | 220 (9.38) | 344 (14.74) | |
| 3. About every week | 203 (8.65) | 300 (12.85) | |
| 4. About every month | 456 (19.44) | 468 (20.05) | |
| 5. Rarely or never | 1225 (52.22) | 831 (35.6) | |
| who5_1 |  |  | |
| 1. At no time | 43 (1.83) | 47 (2.01) | |
| 2. Some of the time | 189 (8.06) | 302 (12.94) | |
| 3. Less than half of the time | 219 (9.34) | 378 (16.2) | |
| 4. More than half of the time | 603 (25.7) | 797 (34.15) | |
| 5. Most of the time | 1034 (44.08) | 699 (29.95) | |
| 6. All the time | 258 (11) | 111 (4.76) | |
| who5_2 |  |  | |
| 1. At no time | 80 (3.41) | 162 (6.94) | |
| 2. Some of the time | 274 (11.68) | 517 (22.15) | |
| 3. Less than half of the time | 339 (14.45) | 561 (24.04) | |
| 4. More than half of the time | 655 (27.92) | 593 (25.41) | |
| 5. Most of the time | 754 (32.14) | 424 (18.17) | |
| 6. All the time | 244 (10.4) | 77 (3.3) | |
| who5_3 |  |  | |
| 1. At no time | 90 (3.84) | 230 (9.85) | |
| 2. Some of the time | 268 (11.42) | 548 (23.48) | |
| 3. Less than half of the time | 379 (16.16) | 573 (24.55) | |
| 4. More than half of the time | 663 (28.26) | 562 (24.08) | |
| 5. Most of the time | 666 (28.39) | 321 (13.75) | |
| 6. All the time | 280 (11.94) | 100 (4.28) | |
| who5_4 |  |  | |
| 1. At no time | 352 (15) | 631 (27.04) | |
| 2. Some of the time | 510 (21.74) | 706 (30.25) | |
| 3. Less than half of the time | 444 (18.93) | 455 (19.49) | |
| 4. More than half of the time | 479 (20.42) | 326 (13.97) | |
| 5. Most of the time | 408 (17.39) | 173 (7.41) | |
| 6. All the time | 153 (6.52) | 43 (1.84) | |
| who5_5 |  |  | |
| 1. At no time | 105 (4.48) | 188 (8.05) | |
| 2. Some of the time | 342 (14.58) | 591 (25.32) | |
| 3. Less than half of the time | 351 (14.96) | 417 (17.87) | |
| 4. More than half of the time | 579 (24.68) | 551 (23.61) | |
| 5. Most of the time | 669 (28.52) | 450 (19.28) | |
| 6. All the time | 300 (12.79) | 137 (5.87) | |
| famdec |  |  |  |
| 1. Very strongly disagree | 111 (4.35) | 139 (5.39) |  |
| 2. 2 | 97 (3.8) | 175 (6.79) |  |
| 3. 3 | 112 (4.39) | 229 (8.89) |  |
| 4. 4 | 231 (9.04) | 271 (10.52) |  |
| 5. 5 | 330 (12.92) | 356 (13.81) |  |
| 6. 6 | 387 (15.15) | 383 (14.86) |  |
| 7. Very strongly agree | 1286 (50.35) | 1024 (39.74) |  |
| famhelp |  |  |  |
| 1. Very strongly disagree | 101 (3.95) | 111 (4.31) |  |
| 2. 2 | 96 (3.76) | 156 (6.05) |  |
| 3. 3 | 98 (3.84) | 233 (9.04) |  |
| 4. 4 | 204 (7.99) | 299 (11.6) |  |
| 5. 5 | 304 (11.9) | 364 (14.12) |  |
| 6. 6 | 370 (14.49) | 365 (14.16) |  |
| 7. Very strongly agree | 1381 (54.07) | 1049 (40.71) |  |
| famsup |  |  |  |
| 1. Very strongly disagree | 138 (5.4) | 173 (6.71) |  |
| 2. 2 | 142 (5.56) | 248 (9.62) |  |
| 3. 3 | 158 (6.19) | 298 (11.56) |  |
| 4. 4 | 259 (10.14) | 285 (11.06) |  |
| 5. 5 | 355 (13.9) | 341 (13.23) |  |
| 6. 6 | 391 (15.31) | 426 (16.53) |  |
| 7. Very strongly agree | 1111 (43.5) | 806 (31.28) |  |
| famtalk |  |  |  |
| 1. Very strongly disagree | 180 (7.05) | 257 (9.97) |  |
| 2. 2 | 184 (7.2) | 301 (11.68) |  |
| 3. 3 | 217 (8.5) | 304 (11.8) |  |
| 4. 4 | 272 (10.65) | 340 (13.19) |  |
| 5. 5 | 335 (13.12) | 333 (12.92) |  |
| 6. 6 | 341 (13.35) | 300 (11.64) |  |
| 7. Very strongly agree | 1025 (40.13) | 742 (28.79) |  |
| friendcounton |  |  |  |
| 1. Very strongly disagree | 133 (5.21) | 109 (4.23) |  |
| 2. 2 | 165 (6.46) | 190 (7.37) |  |
| 3. 3 | 232 (9.08) | 245 (9.51) |  |
| 4. 4 | 388 (15.19) | 335 (13) |  |
| 5. 5 | 485 (18.99) | 431 (16.72) |  |
| 6. 6 | 416 (16.29) | 469 (18.2) |  |
| 7. Very strongly agree | 735 (28.78) | 798 (30.97) |  |
| friendhelp |  |  |  |
| 1. Very strongly disagree | 129 (5.05) | 83 (3.22) |  |
| 2. 2 | 161 (6.3) | 143 (5.55) |  |
| 3. 3 | 234 (9.16) | 231 (8.96) |  |
| 4. 4 | 419 (16.41) | 322 (12.5) |  |
| 5. 5 | 512 (20.05) | 486 (18.86) |  |
| 6. 6 | 416 (16.29) | 476 (18.47) |  |
| 7. Very strongly agree | 683 (26.74) | 836 (32.44) |  |
| friendshare |  |  |  |
| 1. Very strongly disagree | 123 (4.82) | 91 (3.53) |  |
| 2. 2 | 130 (5.09) | 121 (4.7) |  |
| 3. 3 | 162 (6.34) | 149 (5.78) |  |
| 4. 4 | 284 (11.12) | 261 (10.13) |  |
| 5. 5 | 432 (16.91) | 362 (14.05) |  |
| 6. 6 | 450 (17.62) | 483 (18.74) |  |
| 7. Very strongly agree | 973 (38.1) | 1110 (43.07) |  |
| friendtalk |  |  |  |
| 1. Very strongly disagree | 181 (7.09) | 128 (4.97) |  |
| 2. 2 | 186 (7.28) | 204 (7.92) |  |
| 3. 3 | 224 (8.77) | 194 (7.53) |  |
| 4. 4 | 313 (12.26) | 263 (10.21) |  |
| 5. 5 | 399 (15.62) | 364 (14.12) |  |
| 6. 6 | 388 (15.19) | 442 (17.15) |  |
| 7. Very strongly agree | 863 (33.79) | 982 (38.11) |  |
| studaccept |  |  |  |
| 1. Strongly agree | 593 (23.22) | 375 (14.55) |  |
| 2. Agree | 1048 (41.03) | 947 (36.75) |  |
| 3. Neither/nor | 545 (21.34) | 731 (28.37) |  |
| 4. Disagree | 199 (7.79) | 311 (12.07) |  |
| 5. Strongly disagree | 169 (6.62) | 213 (8.27) |  |
| studhelpful |  |  |  |
| 1. Strongly agree | 324 (12.69) | 232 (9) |  |
| 2. Agree | 916 (35.87) | 863 (33.49) |  |
| 3. Neither/nor | 739 (28.94) | 823 (31.94) |  |
| 4. Disagree | 362 (14.17) | 466 (18.08) |  |
| 5. Strongly disagree | 213 (8.34) | 193 (7.49) |  |
| studtogether |  |  |  |
| 1. Strongly agree | 408 (15.97) | 249 (9.66) |  |
| 2. Agree | 1003 (39.27) | 876 (33.99) |  |
| 3. Neither/nor | 731 (28.62) | 1005 (39) |  |
| 4. Disagree | 222 (8.69) | 257 (9.97) |  |
| 5. Strongly disagree | 190 (7.44) | 190 (7.37) |  |
| teacheraccept |  |  |  |
| 1. Strongly agree | 408 (15.97) | 309 (11.99) |  |
| 2. Agree | 1151 (45.07) | 1051 (40.78) |  |
| 3. Neither/nor | 611 (23.92) | 839 (32.56) |  |
| 4. Disagree | 237 (9.28) | 247 (9.58) |  |
| 5. Strongly disagree | 147 (5.76) | 131 (5.08) |  |
| teachercare |  |  |  |
| 1. Strongly agree | 218 (8.54) | 149 (5.78) |  |
| 2. Agree | 741 (29.01) | 645 (25.03) |  |
| 3. Neither/nor | 955 (37.39) | 1103 (42.8) |  |
| 4. Disagree | 375 (14.68) | 443 (17.19) |  |
| 5. Strongly disagree | 265 (10.38) | 237 (9.2) |  |
| teachertrust |  |  |  |
| 1. Strongly agree | 321 (12.57) | 175 (6.79) |  |
| 2. Agree | 801 (31.36) | 637 (24.72) |  |
| 3. Neither/nor | 743 (29.09) | 899 (34.89) |  |
| 4. Disagree | 353 (13.82) | 504 (19.56) |  |
| 5. Strongly disagree | 336 (13.16) | 362 (14.05) |  |
| selfsupp_1 |  |  |  |
| 1. Never | 47 (1.74) | 45 (1.7) |  |
| 2. Rarely | 233 (8.64) | 389 (14.73) |  |
| 3. Sometimes | 676 (25.06) | 739 (27.98) |  |
| 4. Most of the time | 1258 (46.63) | 1158 (43.85) |  |
| 5. Always | 484 (17.94) | 310 (11.74) |  |
| selfsupp_2 |  |  |  |
| 1. Never | 50 (1.85) | 34 (1.29) |  |
| 2. Rarely | 146 (5.41) | 219 (8.29) |  |
| 3. Sometimes | 642 (23.8) | 748 (28.32) |  |
| 4. Most of the time | 1415 (52.45) | 1325 (50.17) |  |
| 5. Always | 445 (16.49) | 315 (11.93) |  |
| selfsupp_3 |  |  |  |
| 1. Never | 349 (12.94) | 143 (5.41) |  |
| 2. Almost never | 864 (32.02) | 562 (21.28) |  |
| 3. Sometimes | 799 (29.61) | 972 (36.8) |  |
| 4. Fairly often | 499 (18.5) | 705 (26.69) |  |
| 5. Very often | 187 (6.93) | 259 (9.81) |  |
| selfsupp_4 |  |  |  |
| 1. Never | 114 (4.23) | 123 (4.66) |  |
| 2. Almost never | 302 (11.19) | 553 (20.94) |  |
| 3. Sometimes | 769 (28.5) | 1020 (38.62) |  |
| 4. Fairly often | 982 (36.4) | 694 (26.28) |  |
| 5. Very often | 531 (19.68) | 251 (9.5) |  |
| selfsupp_5 |  |  |  |
| 1. Never | 157 (5.82) | 176 (6.66) |  |
| 2. Almost never | 491 (18.2) | 684 (25.9) |  |
| 3. Sometimes | 1090 (40.4) | 1071 (40.55) |  |
| 4. Fairly often | 751 (27.84) | 552 (20.9) |  |
| 5. Very often | 209 (7.75) | 158 (5.98) |  |
| selfsupp_5 |  |  |  |
| 1. Never | 502 (18.61) | 177 (6.7) |  |
| 2. Almost never | 978 (36.25) | 562 (21.28) |  |
| 3. Sometimes | 734 (27.21) | 802 (30.37) |  |
| 4. Fairly often | 328 (12.16) | 672 (25.44) |  |
| 5. Very often | 156 (5.78) | 428 (16.21) |  |
| fasbathroom |  |  |  |
| 1. None | 9 (0.33) | 7 (0.27) |  |
| 2. One | 1558 (57.75) | 1590 (60.2) |  |
| 3. Two | 931 (34.51) | 856 (32.41) |  |
| 4. More than two | 200 (7.41) | 188 (7.12) |  |
| fasbedroom |  |  |  |
| 1. No | 753 (27.91) | 736 (27.87) |  |
| 2. Yes | 1945 (72.09) | 1905 (72.13) |  |
| fascomputers |  |  |  |
| 1. None | 29 (1.07) | 37 (1.4) |  |
| 2. One | 226 (8.38) | 235 (8.9) |  |
| 3. Two | 513 (19.01) | 507 (19.2) |  |
| 4. More than two | 1930 (71.53) | 1862 (70.5) |  |
| fasdishwash |  |  |  |
| 1. No | 526 (19.5) | 563 (21.32) |  |
| 2. Yes | 2172 (80.5) | 2078 (78.68) |  |
| fasfamcar |  |  |  |
| 1. No | 116 (4.3) | 130 (4.92) |  |
| 2. Yes, one | 701 (25.98) | 733 (27.75) |  |
| 3. Yes, two or more | 1881 (69.72) | 1778 (67.32) |  |
| fasholidays |  |  |  |
| 1. Not at all | 984 (36.47) | 992 (37.56) |  |
| 2. Once | 771 (28.58) | 760 (28.78) |  |
| 3. Twice | 442 (16.38) | 396 (14.99) |  |
| 4. More than twice | 501 (18.57) | 493 (18.67) |  |
|  | | | |
