# Supplementary figures and images for: Support system networks: how support systems shape problematic social media use, mental health, and substance use in Czech adolescents
Source: Child Adolesc Psychiatry Ment Health. 2026 Apr 5;20:78. doi: 10.1186/s13034-026-01081-w (PMC13235073; doi:10.1186/s13034-026-01081-w)

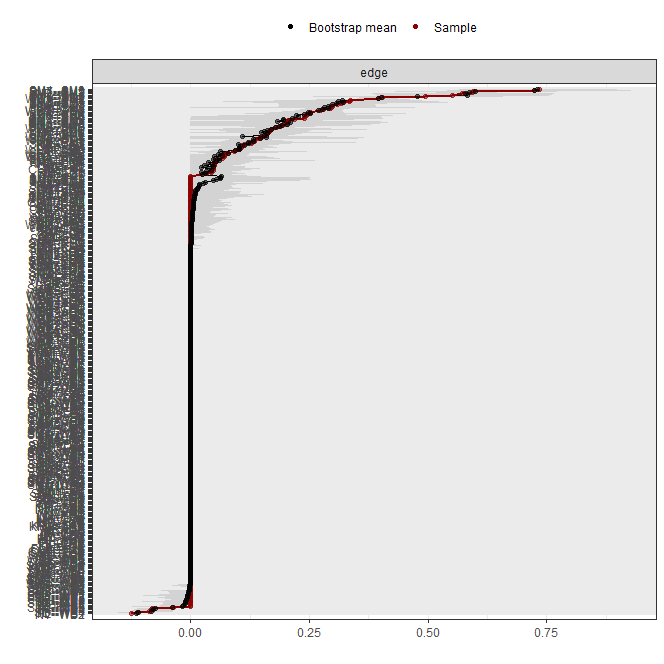

Supplement: Supplementary file 2 — Supplementary Material 2. [file 13034_2026_1081_MOESM2_ESM.png]

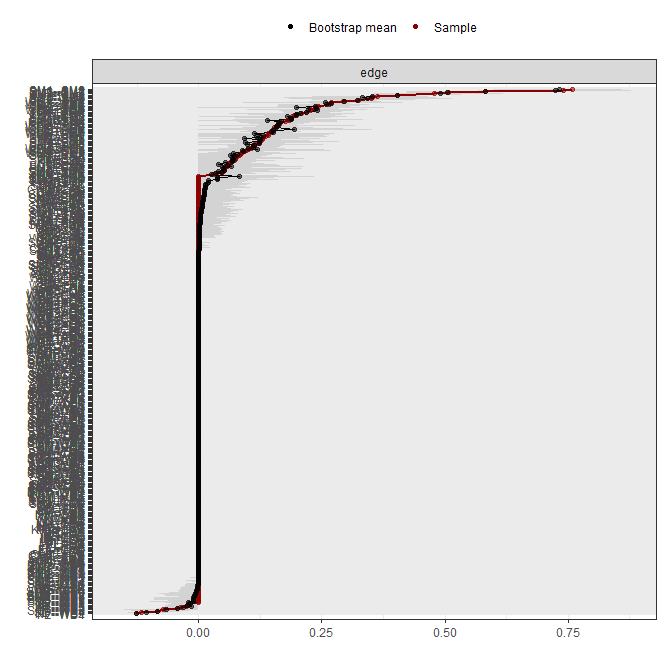

Supplement: Supplementary file 3 — Supplementary Material 3. [file 13034_2026_1081_MOESM3_ESM.png]
